# Supplementary material for: Insecticidal Effect of the Entomopathogenic Fungus Lecanicillium araneicola HK-1 in Aphis craccivora (Hemiptera: Aphididae)
Source: Insects. 2023 Nov 6;14(11):860. doi: 10.3390/insects14110860 (PMC10672225; doi:10.3390/insects14110860)
Supplement: Supplementary file 1 [file insects-14-00860-s001.zip › insects-2639039-supplementary.pdf]

Supplemental materials

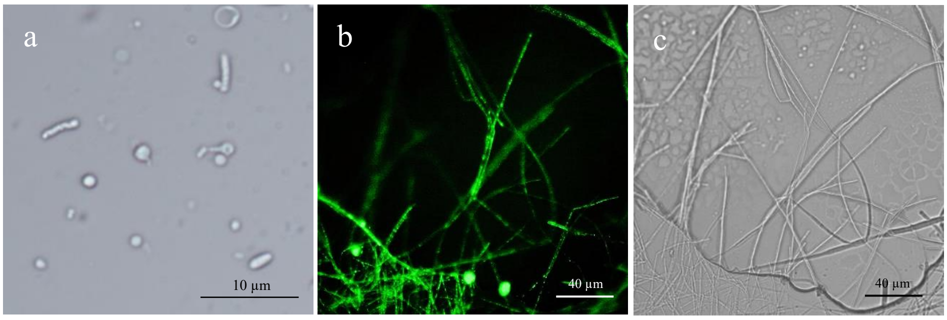

Figure S1. Fluorescence performance of strain HK-1-GFP. **a** Protoplast. **b** Mycelia under blue light. **c** Mycelia under white light.

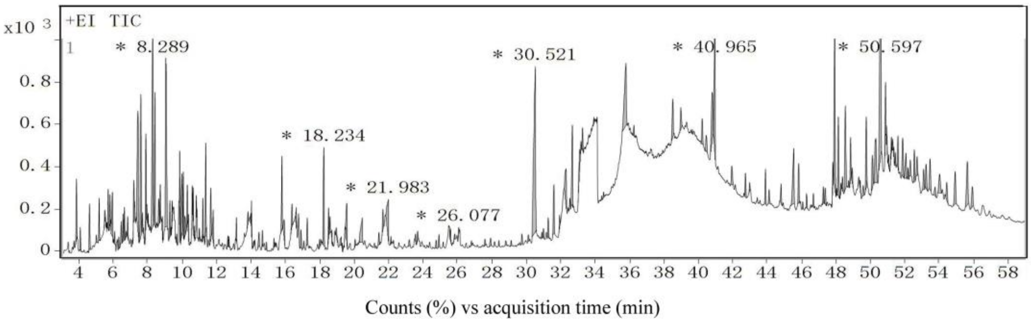

Figure S2. Total ions chromatogram composition of No.3component.

Table S1. Primer sequences used in qRT-PCR.

Gene	Accession no.	Primer sequences (5'-3')
GST-S1	MT130840.1	F: CGACATCACTGCTCTGGCTG<br>R: TGCCAAATGGCATAGTCGGT
GST-D1	MT130836.1	F: GTGACGACTCCGAGCAAAGG<br>R: GCAAATTGCTCGGCTTTCGC
GST-D2	MT130837.1	F: AGATCCGTTTTGCTGACCGC<br>R: CACGATGATTGCACGGCTCT
CarE1	KAF0769975.1	F: AGCGGGCCCATATCTGGAAT<br>R: GTCGATGCGTTCCACACTCC
CarE2	KAF0769984.1	F: GGCGATCTGTTGCTCGTTGA<br>R: ACACACCCACCTTGAAACGG
CarE3	KAF0769982.1	F: CGGTTGCGGTCCACACTATC<br>R: AGAGCTGCCACCTGATCCTT
AChE-X1	KAF0771168.1	F: TTTTCGGTGAATCGGCAGGC<br>R: TCCCGTGACAAAATTGCCCA
RPS8	GAJW01000269.1	F: GTCGTCCGAGCCATTCTTT<br>R: TCCTGTCTTCCTGCGTTTATG

Table S2. The virulence test results of strain HK-1 crude extract on *A.* craccivora adults at 24 h.

Crude extract concentration (mg mL^{-1})	24 h average mortality ± SE (%)
Control	10.00 ± 4.08c
7.5	16.67 ± 6.24c
15	40.00 ± 10.80b
30	53.33 ± 10.27b
60	76.67 ± 4.71a
120	90.00 ± 4.08a

Different little letters in the table indicate significant differences between different treatments ($P < 0.05$).

Table S3. The virulence test results of acetamiprid on *A. craccivora* adults at 48 h.

acetamiprid concentration (μg mL^{-1})	48 h average mortality ± SE (%)
Control	1.67 ± 0.03e
0.5	15.67± 0.03d
1	28.33 ± 0.03d
2	55 ± 0.10c
4	81.67 ± 0.03b
8	85 ± 0.05b
12	95 ± 0.00a

Different little letters in the table indicate significant differences between different treatments ($P < 0.05$).

Table S4 Toxicity of isolated components on *A. craccivora* adults after 24 h

Component (10 mg mL-1)	24 h average mortality ± SE (%)
3	64.44 ± 16.78a
12	44.44 ± 20.37a
Crude extract	13.33 ± 17.64b
Acetone control	6.67 ± 6.67b

Different little letters in the table indicate significant differences between different treatments ($P < 0.05$).

Table S5. Compound composition and percentage content of No. 3 component.

Peak	Rt^a (min)	Compound name	Peak area (%)
4	4.615	2-Pentanone, 4-hydroxy-4-methyl-	0.65
6	5.177	Benzene, 1, 3-dimethyl-	0.51
7	5.507	Cyclohexanol	0.14
8	5.704	Benzene, 1, 3-dimethyl-	0.48
9	5.814	Nonane	0.34
10	5.95	Cyclohexanol, 1-ethyl-	0.80
11	6.077	4-Methyl-1, 6-heptadien-4-ol	0.14
12	6.316	1H-Indene, octahydro-, cis-	0.15
13	6.454	Benzene, (1-methylethyl)-	0.35
14	6.565	Cyclohexane, propyl-	0.34
15	6.641	Octane, 3, 6-dimethyl-	0.42
16	6.75	Acetic acid, methoxy-, ethyl ester	0.07
17	6.832	Heptane, 3-ethyl-2-methyl-	0.28
18	7.187	Benzene, propyl-	0.46

Peak	Rt[a] (min)	Compound name	Peak area (%)
19	7.425	Undecane, 5, 5-dimethyl-	3.10
20	7.6	Benzene, 1-ethyl-3-methyl-	2.37
21	7.686	Cyclohexane, 1-methyl-4-(1-methylethyl)-, trans-	0.23
22	7.749	m-Menthane	0.07
23	7.89	2, 6-Xylidine	1.23
25	8.023	Cyclohexane, 1-methyl-3-propyl-	0.11
26	8.289	Benzenamine, 2-ethyl-	4.24
27	8.418	Decane	1.94
28	8.637	Benzene, (2-methylpropyl)-	0.43
29	8.726	Benzene, 1-methyl-4-propyl-	0.46
30	8.84	Menthyl acetate	0.24
31	9.044	**p-Cymene**	3.91
32	9.286	Cycloh Vanillicexane, butyl-	0.48
33	9.42	Benzene, cyclopropyl-	0.30
34	9.681	Tetrahydro carvone	0.10
35	9.86	Benzene, 1-methyl-3-propyl-	0.76
36	9.99	1, 3, 8-p-Menthatriene	1.27
37	10.067	Benzene, 4-ethyl-1, 2-dimethyl-	0.86
38	10.157	Benzene, 1, 2-diethyl-	0.38
39	10.298	2-Pyridinepropanamide, N-phenyl-	0.49
40	10.365	Sulfurous acid, hexyl nonyl ester	0.19
41	10.591	Benzene, 4-ethyl-1, 2-dimethyl-	0.33
42	10.637	o-Cymene	0.23
43	10.698	Indan, 1-methyl-	0.09
44	10.823	Benzene, 1-ethyl-2, 4-dimethyl-	0.26
46	11.205	Undecane	0.47
47	11.373	Nonanal	1.25
48	11.667	Phenylethyl Alcohol	0.90
49	11.804	2, 4-Dimethyl-2, 4-pentanediol	0.45
50	12.656	Butanoic acid, 1, 1-dimethyl-2-phenylethyl ester	0.11
51	12.724	Benzene, 2-ethyl-1, 4-dimethyl-	0.09
52	13.154	Benzene, 1, 3-dimethoxy-	0.24
53	13.821	Ethanol, 1-(2-butoxyethoxy)-	0.18
54	13.989	Benzoic acid	0.10
55	14.027	Octanoic acid	0.24
56	14.151	Catechol	0.09
57	14.462	1, 3-Octanediol	0.25
58	14.662	Ethanol, 2-phenoxy-	0.19
59	14.899	2-Ethylhexyl acrylate	0.09
60	15.336	2-Sec-Butylcyclohexanone	0.10
61	15.798	2-Decenal, (E)-	1.02
62	15.921	Benzeneacetic acid	0.41
63	16.386	Triacetone triperoxide	0.37

Peak	Rt[a] (min)	Compound name	Peak area (%)
64	16.629	Nonanoic acid	0.17
65	16.904	3-tert-Butyl-2-pyrazolin-5-one	0.30
66	17.073	Formamide, N, N-dibutyl-	0.07
67	17.27	2, 4-Decadienal, (E, E)-	0.39
68	17.996	4, 4, 6-Trimethyl-cyclohex-2-en-1-ol	0.12
69	18.234	Phenol, 2, 6-dimethoxy-	1.77
70	18.512	2-Undecenal	0.38
71	18.595	Propanoic acid, 3-chloro-, 4-formylphenyl ester	0.22
72	18.703	Ethyl hydrogen succinate	0.10
73	18.96	Orcinol	0.16
74	19.245	3-Octanol, 2, 6-dimethyl-	0.17
75	19.295	2, 4-Octadienoic acid, 3-methyl-, methyl ester	0.06
76	19.469	Vanillin	0.07
77	19.569	1-Triazene, 3, 3-dimethyl-1-phenyl-	0.72
78	20.055	1, 4-Benzenediol, 2, 5-dimethyl-	0.11
79	20.47	Isopentyl 3-hydroxy-2-methylenebutanoate	0.86
80	21.435	Benzoic acid, 2-(1-oxopropyl)-, methyl ester	0.20
81	21.667	Ethanone, 1-(3-hydroxy-4-methoxyphenyl)-	0.30
82	21.983	9-Oxononanoic acid	1.13
83	22.128	Benzeneacetic acid, 4-methoxy-	0.08
84	23.198	5-Ethoxy-2-methyl-pyridine	0.11
85	23.564	Dodecanoic acid	0.08
86	23.688	Metacetamol	0.15
87	24.763	Tetrahydrofuran-2-one, 5-[1-hydroxyhexyl]-	0.12
88	24.927	Cyclohepta[c]pyrazole-3-carboxylic acid, 1, 4, 5, 6, 7, 8-hexahydro-	0.19
89	25.599	Tributyl phosphate	0.17
90	25.842	2-n-Heptylfuran	0.07
91	26.077	2-Cyclohexen-1-one, 3, 5, 5-trimethyl-4-(3-oxobutyl)-	0.09
92	27.605	4-(1, 5-Dihydroxy-2, 6, 6-trimethylcyclohex-2-enyl)but-3-en-2-one	0.10
93	27.934	Tetradecanoic acid	0.16
95	28.802	6-Methoxy-3-acetamido-2-picoline	0.07
96	29.12	Tricosane-6, 8-dione	0.08
97	29.743	3-(6-Methoxy-3-methyl-2-benzofuranyl)acrylic acid	0.16
98	30.119	Phthalic acid, hept-4-yl isobutyl ester	0.11
99	30.521	4H-1-Benzopyran-4-one, 5-hydroxy-7-methoxy-2-methyl-	6.17
100	30.903	beta.-Asarone	0.07
101	30.998	1-Phenanthrenecarboxylic acid, 7-ethyltetradecahydro-1, 4a, 7-trimethyl-, methyl ester, [1R-(1.alpha., 4a.beta., 4b.alpha., 7.beta., 8a.beta., 10a.alpha.)]-	0.13
102	31.274	Trispiro[4.2.4.2.4.2.]heneicosane	0.30

Peak	Rt[a] (min)	Compound name	Peak area (%)
103	31.606	Benzo[c]phenanthrene	0.83
104	31.94	Dibutyl phthalate	0.17
106	32.54	4H-1-Benzopyran-4-one, 5, 7-dihydroxy-2-methyl-	0.16
107	32.678	10-Methylanthracene-9-carboxaldehyde	1.47
108	33.115	Dibenzo[b, f][1, 4]diazocine	0.51
111	33.929	9, 12-Octadecadienoic acid (Z, Z)-, methyl ester	0.09
112	34.494	9-Octadecenoic acid, methyl ester, (E)-	0.07
113	35.786	9, 12-Octadecadienoic acid (Z, Z)-	2.55
115	38.514	Benzene, 1-butyl-4-[(4-ethylphenyl)ethynyl]-	0.82
117	40.223	Coniferyl aldehyde, TBDMS derivative	0.44
118	40.459	Quinazoline, 3, 4-dihydro-2-allylthio-4-spirocyclohexane-	0.37
120	40.965	Hymecromone	2.74
124	43.896	Anthracene, 1, 2, 3, 4-tetrahydro-9, 10-dimethyl-	0.49
127	45.538	2, 3-Dihydroxypropyl elaidate	1.50
131	46.686	Olean-13(18)-ene	0.19
132	47.264	Androst-5-en-17-one, 3-hydroxy-16-(phenylmethylene)-	0.22
135	47.926	Anthraergostatetraenol benzoate	3.34
137	48.136	Anthraergostatetraenol hexahydrobenzoate	1.49
150	50.597	Ergosta-5, 7, 9(11), 22-tetraen-3-ol, (3.beta., 22E)-	5.44
151	50.874	9, 19-Cyclolanostan-3-ol, acetate, (3.beta.)-	0.90
153	51.055	Ergosterol	0.14
154	51.217	Lycopene	0.27
156	51.358	Neoergosterol	0.07
160	52.056	Ergosta-4, 6, 8(14), 22-tetraen-3-one	0.61
163	52.548	Stigmasterol, 3, 4-dedihydro-, acetate(ester)	0.53
165	53.092	Oleana-11, 13(18)-diene	0.38
167	53.465	Ergosta-4, 6, 8(14), 22-tetraen-3-one	0.65
172	55.634	3.alpha., 5.alpha.-Cyclo-ergosta-7, 9(11), 22t-triene-6.beta.-ol	1.32
173	55.926	7, 8-Epoxylanostan-11-ol, 3-acetoxy-	0.53

[a] Rt, retention time (min)
